# Telesimulation in Medical Education for High-Acuity Low-Occurrence Procedures and Clinical Encounters for Physicians and Medical Trainees in Emergency Medicine: Protocol for a Systematic Review

**DOI:** 10.2196/53565

**Published:** 2025-05-09

**Authors:** Kurtis W Thornhill, Jennifer Jewer, Queen Jacques, Michael H Parsons

**Affiliations:** 1 Faculty of Medicine Memorial University of Newfoundland St. John's, NL Canada; 2 Faculty of Business Memorial University of Newfoundland St. John's, NL Canada; 3 Discipline of Emergency Medicine Faculty of Medicine Memorial University of Newfoundland St. John's, NL Canada

**Keywords:** assessment, task performance, medical education, emergency medicine, telesimulation, synchronous, asynchronous, medical education, simulation-based, physicians, medical trainees, emergency medicine, systematic review, proficiency, high-acuity low-occurrence, HALO

## Abstract

**Background:**

Proficiency in high-acuity low-occurrence (HALO) procedures and clinical encounters is crucial for physicians and medical trainees in emergency medicine. Simulation-based medical education (SBME) provides valuable learning opportunities for these skills. However, accessing SBME can be challenging. Remotely delivered SBME, known as telesimulation, can enhance access to such training, especially in remote locations.

**Objective:**

Based on this review, the research team aims to evaluate the effectiveness of telesimulation in enhancing learning outcomes of HALO procedures and clinical encounters in emergency medicine.

**Methods:**

A systematic review will be conducted using the electronic databases PubMed, CINAHL, Embase, and Cochrane, focusing on studies published in English from 2011 to the present. The inclusion criteria are emergency physicians and medical trainees as learners; telesimulation sessions where the adequate performance of the procedure or clinical encounter, retention of information, or user feedback after implementing telesimulation were assessed; and original research in the form of a randomized controlled trial or nonrandomized experiments with an intervention and control group and pre- and posttest design. The exclusion criterion is defined as any study that does not fully meet the inclusion criteria. The primary outcome is the effectiveness of telesimulation in enhancing learning outcomes for HALO procedures and clinical encounters for physicians and medical trainees in emergency medicine. The secondary outcomes are the effectiveness of telesimulation for these procedures and clinical encounters delivered asynchronously and synchronously for physicians and medical trainees in emergency medicine. At least two reviewers will conduct data extraction and quality assessment. The primary and secondary outcomes will be analyzed through a systematic narrative synthesis. The methodological quality of comparative studies will be assessed using the Downs and Black checklist. The interrater reliability among the authors will be analyzed with Cohen κ.

**Results:**

This project was funded in the summers of 2022 and 2023 by two Summer Undergraduate Research Awards from the Memorial University of Newfoundland Faculty of Medicine. The literature search and screening will begin in April 2025, and the results of the systematic review will be available in the summer of 2026.

**Conclusions:**

The results of the systematic review could inform the development of research on telesimulation for HALO procedures in emergency medicine. By investigating this topic, more effective telesimulation sessions can be designed in the future, potentially enhancing the skills of physicians and medical trainees.

**International Registered Report Identifier (IRRID):**

PRR1-10.2196/53565

## Introduction

### Background

On-the-job experience is not always adequate in providing emergency physicians with the skills necessary for high-acuity low-occurrence (HALO) procedures and clinical encounters. This is because HALO procedures and clinical encounters may present in an intermittent and episodic nature to emergency departments, making it hard to plan for them. Exacerbated by the fact that these skills are particularly susceptible to degradation over time [1], frequent follow-up training is needed. Increasingly, educators are turning to simulation-based medical education (SBME) to provide physicians and medical trainees with learning opportunities for HALO procedures and clinical encounters [2-4]. SBME has been defined as any educational activity that uses simulative aids to replicate clinical scenarios [5]. SBME provides a learning environment where physicians and medical trainees can learn skills without compromising patient safety [6], and it has also been found to reduce health care costs by improving medical providers’ competencies [5]. However, accessing SBME can be challenging for individuals working in rural or remote locations due to financial, geographical, and time constraints [7,8].

Some of these challenges have been overcome by using technology to remotely deliver this SBME; this is known as telesimulation [9-18]. Telesimulation is a type of SBME that has been defined as the use of telecommunication and simulation resources to provide education, training, assessment, and debriefing to learners at an off-site location [19]. The role of telesimulation is an active area of research in procedural task training, debriefing, and instruction delivery in critical care fields such as emergency medicine [19]. Using telesimulation, the instructor does not need to travel to the learners and vice versa. Other benefits of telesimulation include eliminating time barriers to educational content delivery, allowing for interinstitutional networking/collaboration, and providing a means for rapid dissemination of new content in medical education [19]. Additionally, the resources necessary for telesimulation have been described as free or low-cost [19].

Telesimulation in SBME is typically categorized as either asynchronous or synchronous. Asynchronous instruction involves using prerecorded video demonstrations to teach procedures or manage clinical scenarios and has been shown to be an effective teaching method [9,11]. Synchronous instructions, on the other hand, are delivered live to learners and have been shown to be just as effective as traditional methods [12-18].

### Review Aims

The primary outcome of this study is to determine the effectiveness of telesimulation of HALO procedures and clinical encounters in enhancing learning outcomes for physicians and medical trainees in emergency medicine. Evaluating the effectiveness of telesimulation is important to ensure emergency physicians and medical trainees receive appropriate training for HALO procedures and clinical encounters. This includes, but is not limited to, procedures such as chest tube insertion and surgical airway management, and clinical scenarios such as managing anaphylaxis, ST-elevated myocardial infarction, and cardiac arrest. A systematic review was published in 2012 to evaluate the effectiveness of instruction design features of simulation-based training interventions [20]. Given the advances in technology over the last decade and ongoing research in telesimulation, further investigation is warranted. This is also supported by the ongoing shift to e-learning methods in emergency medicine established by the COVID-19 pandemic [21].

The secondary outcome of our study is to assess the effectiveness of telesimulation of HALO procedures and clinical encounters delivered asynchronously versus synchronously in enhancing learning outcomes for physicians and medical trainees in emergency medicine. One recommendation from the 2012 review was to investigate what simulation characteristics were effective for specific populations under certain conditions [20]. It also suggested focusing on comparing different types of simulations to assess effective design features [20]. Comparing asynchronous with synchronous instructions will explore these recommendations. Additionally, our study will investigate which methods are most effective for emergency medicine, a topic not well studied in the literature.

Our review will serve as a means of updating the literature on the use of telesimulation in emergency medicine since the review by Cook et al [20] over a decade ago. An updated understanding of telesimulation of HALO procedures and clinical encounters in emergency settings will allow for more effective planning of telesimulation to reach physicians and medical trainees in rural and remote areas where access to in-person SBME is limited.

### Research Question

#### Primary Research Question

How does telesimulation for HALO procedures and clinical encounters impact the learning outcomes of emergency physicians and medical trainees?

#### Secondary Research Question

How does telesimulation for HALO procedures and clinical encounters delivered asynchronously versus synchronously impact the learning outcomes of emergency physicians and medical trainees?

## Methods

### Systematic Review Design

We will include articles that focus on the delivery of telesimulation to teach or enhance the skills necessary for HALO procedures and clinical encounters in emergency medicine. Textbox 1 outlines the framework for the systematic review using a Population, Exposure, Comparator, Outcomes (PECO) structure, which is being registered on PROSPERO. It should be noted that “no instructions provided” refers to studies where learners did not receive instructions before participating in the telesimulation.

**Systematic review framework.**

**Population**

- Emergency physicians or medical trainees as learners

**Exposure**

- Use of telesimulation for high-acuity low-occurrence (HALO) procedures and clinical encounters in emergency medicine

**Comparator**

- Use of in-person methods for HALO procedures and clinical encounters in emergency medicine

**Outcomes**

- Primary: ability to meet learning objectives such as performance of the procedure or clinical encounter
- Secondary: ability to retain information and user feedback on the learning experience

**Primary**

- Effectiveness of telesimulation in enhancing learning outcomes of HALO procedures and clinical encounters for physicians and medical trainees in emergency medicine

**Secondary**

- Effectiveness of telesimulation delivered asynchronously versus synchronously in enhancing learning outcomes of HALO procedures and clinical encounters for physicians and medical trainees in emergency medicine

**Design (study)**

- Eligible studies will include randomized controlled trials and nonrandomized experiments with intervention and control groups with a pre- and posttest design

**Time range**

- 2011 to present day

### Search Strategy

The online databases to be used for the systematic review include CINAHL, Cochrane, Embase, and PubMed. MEDLINE will not be searched given it shares the same material as PubMed. The search strategy was developed by the project team and peer-reviewed by two medical education librarians with expertise in systematic reviews. Literature search strategies will use Medical Subject Headings (MeSH) and key concepts related to simulation-based training for emergency physicians and medical students. This includes “telesimulation,” “medical education,” “task performance,” “assessment,” and “emergency medicine.” Common synonyms and alternative spellings of these concepts will be included in the search strategy.

The search will be limited to the English language. To ensure literature saturation, two researchers will review the cited references of all included articles. PROSPERO will be searched for ongoing or recently completed systematic reviews. As studies relevant to our project are identified, reviewers will assess for additional relevant cited and citing articles. Additionally, key studies identified by the team will be searched for in the results to ensure the validity of the search strategy.

An example of the search strategy for PubMed is demonstrated in Table 1. A similar strategy will be used for each database (Multimedia Appendix 1).

**Table 1:** PubMed search strategy.

Search number	Query
1	((((“Simulation Training”[Mesh] OR “High Fidelity Simulation Training”[Mesh]) OR “Patient Simulation”[Mesh]) OR “Computer Simulation”[Mesh]) OR “Computer-Assisted Instruction”[Mesh]) OR “Educational Technology”[Mesh]
2^a^	simulation OR telesimulation
3	#1 OR #2
4	((“Education, Continuing”[Mesh]) OR “Education, Medical”[Mesh]) OR “Health Personnel/education”[Mesh:NoExp]
5^a^	“medical education” OR “continuing education” OR “virtual learning” OR “distance learning”
6	#4 OR #5
7	“Task Performance and Analysis”[Mesh]
8^a^	“technical skills” OR “technical skill” OR “task performance” OR ((procedure OR procedural OR procedures) AND train*) OR resuscitat*
9	#7 OR #8
10	(((“Educational Measurement”[Mesh:NoExp]) OR “Professional Competence”[Mesh]) OR “Comparative Effectiveness Research”[Mesh]) OR “Program Evaluation”[Mesh]
11^a^	assessment OR feedback OR evaluation OR measurement
12	#10 OR #11
13^a^	(“Emergency Medicine”[Mesh]) OR “Critical Care”[Mesh] OR “emergency medicine” OR “critical Care” OR emergen*
14	#3 AND #6 AND #9 AND #12 AND #13

^a^All fields searched.

### Identification of Studies

The systematic review will follow the standard PRISMA (Preferred Reporting Items for Systematic Reviews and Meta-Analyses) guideline study identification process [22]. This 2-stage process will start with a review of abstracts identified by the search strategy. At least two reviewers will independently screen titles and abstracts filtered by the inclusion criteria of the search. Any disagreement will be resolved through discussion, and the reasons for excluding literature will be documented. In the case that consensus is unable to be reached via discussion, a third researcher on the project team will review the study to make a final decision.

Literature search results will be uploaded to Covidence software. This software will be used to facilitate collaboration among reviewers throughout the study selection process. All team members involved with the review will receive training with the Covidence software before the start of the review.

### Abstract Review

The initial review will examine the abstracts of the articles returned in the searches. To be relevant, an abstract must identify the use of some telesimulation component and involve HALO procedures and clinical encounters relevant to the field of emergency medicine.

Researchers will err on the side of inclusion. If an abstract does not clearly meet a requirement but seems related to the theme, it will be selected for full-text review. If a literature review (eg, narrative, scoping, or systematic) is identified by the search that seems applicable to the research question, the researchers will read the review to determine if any cited studies are relevant to include.

**Figure 1:**
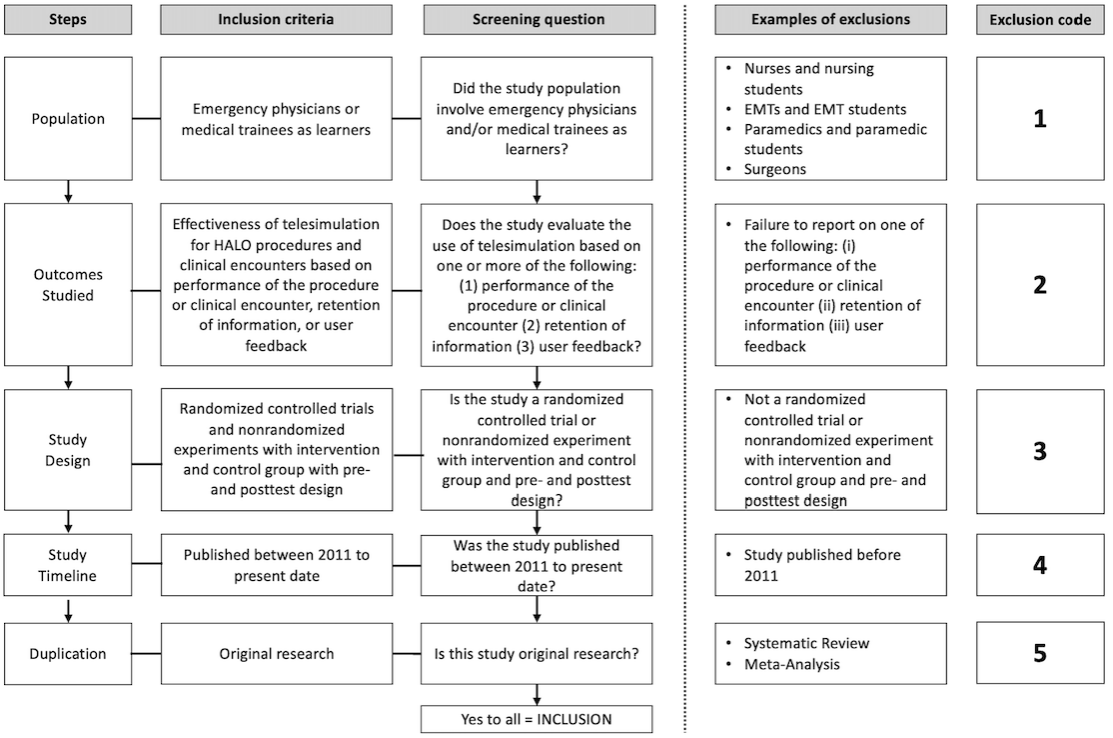
Full-text inclusion screening tool (adapted from Elzinga et al [23]). EMT: emergency medical technician; HALO: high-acuity low-occurrence.

After relevant abstracts are identified, a full-text review will be completed to identify which studies meet all inclusion criteria. Researchers will use a standardized screening tool, based on the tool from Elzinga et al [23], to determine the inclusion eligibility of every study. The tool consists of four questions, each addressing a specific inclusion/exclusion criterion. The first question addresses the population and whether the study involves emergency physicians and medical trainees as learners. The second question focuses on the outcome studied. This includes whether the SBME session in the study evaluated adequate performance of the procedure or clinical encounter, retention of information, or user feedback after implementing telesimulation. Questions three and four focus on the methodology of the paper, which must be original research that is either a randomized controlled trial or nonrandomized experiment with an intervention and control group and pre- and posttest design. This screening tool is illustrated in Figure 1 [23].

### Data Extraction and Synthesis

Data extraction and quality assessment will be completed by at least two reviewers. Both reviewers will perform these tasks independently and then compare results, and any disagreements will be resolved through discussion. In the case that consensus is not met through discussion, a third researcher on the project team will review the article to make a final decision.

Review authors will use standardized data collection forms to ensure consistency while extracting data from reports. Data will be extracted into Microsoft Excel (version 16.96). This is included in Multimedia Appendix 2. The data extracted will include four themes (study characteristics, population characteristics, outcomes, and quality). Textbox 2 summarizes the themes within each.

**Data extraction variables.**

**Study characteristics**

- Publication date, publication author, publication journal, study methodology, study geographic location, study time frame

**Population characteristics**

- Sample size, education level (medical student, resident, or staff physician), preexisting knowledge/skill level of high-acuity low-occurrence procedure or clinical encounter

**Outcomes**

- Primary: performance of the procedure or clinical encounter, retention of information, user feedback
- Secondary: comparison of primary outcomes for asynchronous versus synchronous telesimulation

**Quality**

- Downs and Black [24] checklist

### Quality Assessment

The Downs and Black [24] checklist will be used to assess the methodological quality of the comparative studies. The checklist is comprised of 27 items addressing reporting, external validity, internal validity, and power. Recent systematic reviews have used the following cut points to categorize studies by quality: excellent (26-28), good (20-25), fair (15-19), and poor (≤14) [25,26].

A standardized process will be implemented to ensure consistent understanding among authors on how to proceed with the abstract review, full-text review, data extraction, risk of bias, and Downs and Black [24] checklist. First, all authors will receive training on how to complete each step of the study. Then, authors will complete 10-20 papers and compare results. The interrater reliability will be analyzed with Cohen κ. Authors may proceed with the rest of the papers independently once very good agreement is achieved, defined by κ>0.80 [27]. If this is not achieved, the authors will go through retraining, and a new Fleiss κ will be measured. A final Cohen κ value will be calculated for author agreement at the abstract review, full-text review, data extraction, risk of bias, and Downs and Black [24] checklist stages. This value will be reported in the study results.

### Data Analysis

The primary and secondary outcomes will be analyzed using a systematic narrative synthesis. The narrative synthesis will summarize and explain findings from studies filtered by the inclusion criteria. It will compare reports on the performance of the procedure or clinical encounter as well as the retention of information and user feedback within and between the included studies.

## Results

This project was funded in the summers of 2022 and 2023 from two Summer Undergraduate Research Awards by Memorial University of Newfoundland’s Faculty of Medicine, supporting preliminary literature search and the development of the research proposal and protocol. Additional funding was awarded in March 2025 through the Medical Education Research Fund Award from Memorial University of Newfoundland to support the systematic review.

The literature search and screening process is expected to begin in July 2025. An initial search conducted in July 2023 across four databases identified 3346 studies that met the search criteria. After exporting the citations to Covidence, a systematic review platform, 572 duplicates were removed, leaving 2759 articles for screening. Before screening, an additional search will be conducted to update the identified articles and ensure they are the most current. Title and abstract screening is scheduled to begin in July 2025, with two independent reviewers (KWT and QJ). Abstract screening and full-text review are expected to be completed by the end of October 2025. Data analysis will take place once the full texts have been approved by two independent reviewers (KWT and QJ). The two reviewers will extract all relevant data and key findings, and any discrepancies will be resolved with the principal investigator of the study (MHP). Results of this systematic review are expected to be available by the winter of 2026, aiming to complete the final paper by the summer of 2026.

## Discussion

### Expected Findings

SBME equips emergency physicians and medical trainees with the necessary skills to handle HALO procedures and clinical encounters [2-4]. Without SBME, these skills may be inadequately developed and maintained [1]. However, accessing SBME can be challenging due to financial, geographical, and time constraints [7,8], especially for those based in rural or remote areas [7,8]. Telesimulation has been shown to address these challenges and is as effective as traditional methods [9-18]. We anticipate that telesimulation for teaching HALO procedures and clinical encounters will be just as effective as other SBME methods. We also anticipate that synchronous telesimulation will be demonstrated as more effective than asynchronous telesimulation.

The method of delivering instructions in SBME has been widely studied [19]. Telesimulation is a popular approach using either synchronous or asynchronous instructions [9-18]. Given the advances in technology and the recent shift to internet-based learning due to the COVID-19 pandemic, further investigation into the effectiveness of telesimulation is warranted.

### Limitations

Although many established HALO procedures and clinical encounters exist, their definition appears unclear [28]. Therefore, the selection of specific procedures and clinical encounters for inclusion or exclusion will be guided by those essential for training emergency physicians in Canada and further refined based on the search results. Additionally, other medical specialties beyond emergency medicine have published studies on using telesimulation in SBME for HALO procedures and clinical encounters. Therefore, this report may draw on studies conducted by specialists in other fields involving medical trainees as learners, provided they are relevant and applicable to emergency medicine, particularly if the search does not yield a sufficient number of studies for a comprehensive review.

This protocol serves as a guide for developing the scoping review but may not capture all relevant literature. Studies indexed in databases not included in the searches could be missed. Additionally, restricting the study’s inclusion criteria to publications in English may exclude some relevant works. Despite these limitations, the review will provide key findings in the field and effectively address the proposed objectives.

### Conclusion

This report describes the systematic review protocol that will be used to evaluate the effectiveness of telesimulation to enhance learning outcomes associated with HALO procedures and clinical encounters in emergency medicine. The timeline of this review will include studies published from 2011 to the present. The findings of our review can lead to the design and planning of more effective telesimulation sessions in the future, potentially enhancing the skills of practitioners and medical trainees.

## Appendix

Multimedia Appendix 1 Search strategies for databases.

Multimedia Appendix 2 Data extraction tool.

Multimedia Appendix 3 PRISMA-P (Preferred Reporting Items for Systematic Review and Meta-Analysis Protocols) 2015 checklist.

## Abbreviations

HALO: high-acuity low-occurrence
MeSH: Medical Subject Headings
PECO: Population, Exposure, Comparator, Outcomes
PRISMA: Preferred Reporting Items for Systematic Reviews and Meta-Analyses
SBME: simulation-based medical education
