# Supplementary material for: Telesimulation in Medical Education for High-Acuity Low-Occurrence Procedures and Clinical Encounters for Physicians and Medical Trainees in Emergency Medicine: Protocol for a Systematic Review
Source: JMIR Res Protoc. 2025 May 9;14:e53565. doi: 10.2196/53565 (PMC12102622; doi:10.2196/53565)
Supplement: Multimedia Appendix 1 [file resprot_v14i1e53565_app1.pdf]

CINAHL Search String

Tuesday, July 18, 2023 2:33:35 PM

#	Query	Limiters/Expanders	Last Run Via	Results
S17	S3 AND S6 AND S9 AND S12 AND S15	Limiters - Published Date: 20220101-20231231<br>Expanders - Apply equivalent subjects<br>Search modes - Boolean/Phrase	Interface - EBSCOhost Research Databases<br>Search Screen - Advanced Search<br>Database - CINAHL Plus with Full Text	8
S16	S3 AND S6 AND S9 AND S12 AND S15	Expanders - Apply equivalent subjects<br>Search modes - Boolean/Phrase	Interface - EBSCOhost Research Databases<br>Search Screen - Advanced Search<br>Database - CINAHL Plus with Full Text	141
S15	S13 OR S14	Expanders - Apply equivalent subjects<br>Search modes - Boolean/Phrase	Interface - EBSCOhost Research Databases<br>Search Screen - Advanced Search<br>Database - CINAHL Plus with Full Text	327,142
S14	"emergency medicine" OR "critical Care" OR emergen*	Expanders - Apply equivalent subjects<br>Search modes - Boolean/Phrase	Interface - EBSCOhost Research Databases<br>Search Screen - Advanced Search<br>Database - CINAHL Plus with Full Text	320,901
S13	(MH "Emergency Medicine") OR (MH "Critical Care+")	Expanders - Apply equivalent subjects<br>Search modes - Boolean/Phrase	Interface - EBSCOhost Research Databases<br>Search Screen - Advanced Search<br>Database - CINAHL Plus with Full Text	45,434
S12	S10 OR S11	Expanders - Apply equivalent subjects<br>Search modes - Boolean/Phrase	Interface - EBSCOhost Research Databases<br>Search Screen - Advanced Search	1,975,470

			Database - CINAHL Plus with Full Text	
S11	assessment OR feedback OR evaluation OR measurement	Expanders - Apply equivalent subjects<br>Search modes - Boolean/Phrase	Interface - EBSCOhost Research Databases<br>Search Screen - Advanced Search<br>Database - CINAHL Plus with Full Text	1,923,103
S10	(MH "Educational Measurement") OR (MH "Professional Competence+") OR (MH "Program Evaluation")	Expanders - Apply equivalent subjects<br>Search modes - Boolean/Phrase	Interface - EBSCOhost Research Databases<br>Search Screen - Advanced Search<br>Database - CINAHL Plus with Full Text	138,168
S9	S7 OR S8	Expanders - Apply equivalent subjects<br>Search modes - Boolean/Phrase	Interface - EBSCOhost Research Databases<br>Search Screen - Advanced Search<br>Database - CINAHL Plus with Full Text	85,704
S8	"technical skills" OR "technical skill" OR "task performance" OR ((procedure OR procedural OR procedures) AND train*) OR resuscitat*	Expanders - Apply equivalent subjects<br>Search modes - Boolean/Phrase	Interface - EBSCOhost Research Databases<br>Search Screen - Advanced Search<br>Database - CINAHL Plus with Full Text	82,656
S7	(MH "Task Performance and Analysis+")	Expanders - Apply equivalent subjects<br>Search modes - Boolean/Phrase	Interface - EBSCOhost Research Databases<br>Search Screen - Advanced Search<br>Database - CINAHL Plus with Full Text	21,983
S6	S4 OR S5	Expanders - Apply equivalent subjects<br>Search modes - Boolean/Phrase	Interface - EBSCOhost Research Databases<br>Search Screen - Advanced Search<br>Database - CINAHL Plus with Full Text	104,333

S5	"medical education" OR "continuing education" OR "virtual learning" OR "distance learning"	Expanders - Apply equivalent subjects<br>Search modes - Boolean/Phrase	Interface - EBSCOhost Research Databases<br>Search Screen - Advanced Search<br>Database - CINAHL Plus with Full Text	63,880
S4	(MH "Education, Continuing+") OR (MH "Education, Medical+") OR (MH "Health Personnel/ED")	Expanders - Apply equivalent subjects<br>Search modes - Boolean/Phrase	Interface - EBSCOhost Research Databases<br>Search Screen - Advanced Search<br>Database - CINAHL Plus with Full Text	77,655
S3	S1 OR S2	Expanders - Apply equivalent subjects<br>Search modes - Boolean/Phrase	Interface - EBSCOhost Research Databases<br>Search Screen - Advanced Search<br>Database - CINAHL Plus with Full Text	71,645
S2	simulation OR telesimulation	Expanders - Apply equivalent subjects<br>Search modes - Boolean/Phrase	Interface - EBSCOhost Research Databases<br>Search Screen - Advanced Search<br>Database - CINAHL Plus with Full Text	62,493
S1	(MH "Computer Simulation") OR (MH "Patient Simulation") OR (MH "Computer Assisted Instruction") OR (MH "Educational Technology")	Expanders - Apply equivalent subjects<br>Search modes - Boolean/Phrase	Interface - EBSCOhost Research Databases<br>Search Screen - Advanced Search<br>Database - CINAHL Plus with Full Text	32,797

Cochrane & PubMed Search String

An official website of the United States government
Here's how you know

PubMed Advanced Search Builder

PubMed®

Filters applied: From 2022 to 2023, English. *Clear all*

Add terms to the query box

All Fields

Enter a search term

ADD

Show Index

Query box

Enter / edit your search query here

Search

History and Search Details

Search	Actions	Details	Query	Results	Time
#16	•••		Search: **#3 AND #6 AND #9 AND #12 AND #13** Filters: **English, from 2022 - 2023**	250	07:43:35
#15	•••		Search: **#3 AND #6 AND #9 AND #12 AND #13** Filters: **from 2022 - 2023**	252	07:43:01
#14	•••		Search: **#3 AND #6 AND #9 AND #12 AND #13**	2,129	07:42:19
#13	•••		Search: **("Emergency Medicine"[Mesh]) OR "Critical Care"[Mesh] OR "emergency medicine" OR "critical Care" OR emergen***	983,579	07:41:54
#12	•••		Search: **#10 OR #11**	10,464,333	07:41:34
#11	•••		Search: **assessment OR feedback OR evaluation OR measurement**	10,393,430	07:41:13
#10	•••		Search: **((("Educational Measurement"[Mesh:NoExp]) OR "Professional Competence"[Mesh]) OR "Comparative Effectiveness Research"[Mesh]) OR "Program Evaluation"[Mesh]**	237,782	07:41:02
#9	•••		Search: **#7 OR #8**	600,848	07:40:41
#8	•••		Search: **"technical skills" OR "technical skill" OR "task performance" OR ((procedure OR procedural OR procedures) AND train*) OR resuscitat***	595,867	07:34:37
#7	•••		Search: **"Task Performance and Analysis"[Mesh]**	38,509	07:34:11
#6	•••		Search: **#4 OR #5**	329,325	07:34:00

Search	Actions	Details	Query	Results	Time
#5	•••		Search: **"medical education" OR "continuing education" OR "virtual learning" OR "distance learning"**	259,083	07:33:25
#4	•••		Search: **(("Education, Continuing"[Mesh]) OR "Education, Medical"[Mesh]) OR "Health Personnel/education"[Mesh:NoExp]**	229,553	07:33:03
#3	•••		Search: **#1 OR #2**	970,997	07:32:42
#2	•••		Search: **simulation OR telesimulation**	863,590	07:32:23
#1	•••		Search: **(((("Simulation Training"[Mesh] OR "High Fidelity Simulation Training"[Mesh]) OR "Patient Simulation"[Mesh]) OR "Computer Simulation"[Mesh]) OR "Computer-Assisted Instruction"[Mesh]) OR "Educational Technology"[Mesh]**	420,107	07:31:59

Showing 1 to 16 of 16 entries

FOLLOW NCBI

Connect with NLM

National Library of Medicine
8600 Rockville Pike
Bethesda, MD 20894

Web Policies
FOIA
HHS Vulnerability Disclosure

Help
Accessibility
Careers

NLM NIH HHS USA.gov

Embase Search String

History Save | Delete | Print view | Export | Email Combine using And Or Collapse

#16	#3 AND #6 AND #9 AND #12 AND #13 AND [2022-2023]/py AND [english]/lim	194
#15	#3 AND #6 AND #9 AND #12 AND #13 AND [2022-2023]/py	195
#14	#3 AND #6 AND #9 AND #12 AND #13	1,584
#13	#10 OR #11	6,895,437
#12	**'emergency medicine'**/exp OR **'intensive care'**/exp OR **'emergency medicine'** OR **'critical care'** OR **emergen***	2,217,640
#11	**assessment** OR **feedback** OR **evaluation** OR **measurement**	6,783,729
#10	**'professional competence'**/exp OR **'comparative effectiveness'**/exp OR **'program evaluation'**/exp	211,868
#9	#7 OR #8	537,042
#8	**'technical skills'** OR **'technical skill'** OR **'task performance'** OR ((**procedure** OR **procedural** OR **procedures**) AND **train***) OR **resuscitat***	537,042
#7	**'task performance'**/exp	158,606
#6	#4 OR #5	539,826
#5	**'medical education'** OR **'continuing education'** OR **'virtual learning'** OR **'distance learning'**	428,337
#4	**'continuing education'**/exp OR **'medical education'**/exp	419,353
#3	#1 OR #2	584,793
#2	**simulation** OR **telesimulation**	559,939
#1	**'simulation training'**/exp OR **'patient simulation'**/exp OR **'computer simulation'**/exp OR **'educational technology'**/exp	178,472
